# Exploratory Spinopelvic Compensation Patterns Are Associated with a Strict Composite 12-Month MCID Endpoint After Total Knee Arthroplasty: A Retrospective Risk-Stratification Study

**DOI:** 10.3390/medicina62081481

**Published:** 2026-08-01

**Authors:** İbrahim Altun, Muhammed Melez

**Affiliations:** 1 Department of Orthopedics and Traumatology, Kayseri City Hospital SUAM, University of Health Sciences, 38080 Kayseri, Türkiye; 2 Department of Orthopedics and Traumatology, Memorial Health Group Kayseri Hospital, 38080 Kayseri, Türkiye; muhammed.melez@memorial.com.tr

**Keywords:** total knee arthroplasty, spinopelvic alignment, lumbar lordosis, pelvic tilt, patient-reported outcome measures, MCID, calibration, decision curve analysis, risk stratification

## Abstract

*Background and Objectives:* This study evaluated whether exploratory spinopelvic compensation patterns are associated with 12-month patient-reported recovery after total knee arthroplasty (TKA), with explicit separation between baseline-only prediction and contemporaneous association-based risk stratification. *Materials and Methods:* This retrospective single-center cohort included 700 complete cases selected from 723 primary TKA records after 23 exclusions for incomplete key radiographic alignment data and/or unavailable 12-month patient-reported outcome measures (PROMs). The primary endpoint was a strict composite MCID-based endpoint, defined as failure to achieve the prespecified minimal clinically important difference (MCID) for either the Oxford Knee Score (OKS) or WOMAC at 12 months. OKS- and WOMAC-specific MCID failures were reported as component sensitivity outcomes. K-means clustering was used to define exploratory compensation patterns rather than validated preoperative phenotypes. Predictor timing was separated into a baseline-only model and a contemporaneous model that additionally used 12-month alignment-change variables. *Results:* The strict composite MCID endpoint occurred in 537/700 patients (76.7%). Exploratory patterns showed increasing endpoint prevalence: Pattern A, 153/215 (71.2%); Pattern B, 195/258 (75.6%); and Pattern C, 189/227 (83.3%). The baseline-only ridge logistic model showed moderate discrimination (out-of-fold AUC 0.715, 95% CI 0.670–0.758). The contemporaneous model showed higher internal discrimination (out-of-fold AUC 0.864, 95% CI 0.834–0.894), but it included alignment changes measured in the same follow-up window as the outcome. *Conclusions:* Baseline-only prediction was modest, whereas stronger model performance was observed only in a contemporaneous association model. The exploratory patterns should not be interpreted as stable clinical phenotypes or used for individual-level decisions without external validation.

## 1. Introduction

Total knee arthroplasty (TKA) provides meaningful pain relief and functional improvement for many patients with end-stage knee osteoarthritis. Nevertheless, a clinically important subgroup continues to report persistent symptoms, functional limitation, or dissatisfaction despite technically successful surgery [1,2,3]. This residual symptom burden motivates efforts to identify patients at risk for limited recovery and to understand the mechanisms underlying heterogeneous recovery trajectories.

Recovery after TKA is not solely a local knee phenomenon. Sagittal alignment, pelvic position, lumbar lordosis, and lower-limb alignment may interact as a functional kinetic chain, and several studies have described changes in spinopelvic parameters after TKA or in relation to knee osteoarthritis [4,5,6,7,8,9,10,11,12,13,14,15]. These observations suggest that patients may differ in their capacity for upstream compensation after correction of lower-limb deformity.

Machine learning models have increasingly been used to study arthroplasty outcomes, but many are limited by overfitting, inadequate calibration assessment, or insufficient clarity about predictor timing [16,17,18,19]. This issue is especially important when postoperative alignment changes are measured during the same follow-up window as patient-reported outcome measures (PROMs). Such variables can support association-based stratification, but they should not be described as prospective predictors of future 12-month recovery.

The primary aim was to evaluate whether spinopelvic compensation patterns are associated with a strict composite 12-month MCID endpoint after TKA. A secondary aim was to compare a conservative baseline-only prediction model with a contemporaneous association model that incorporated 12-month alignment changes. Because postoperative alignment-change variables are measured in the same follow-up window as PROM outcomes, the latter model was intended to describe recovery heterogeneity rather than to make prospective preoperative predictions.

## 2. Materials and Methods

### 2.1. Study Design and Reporting

This retrospective cohort study followed STROBE reporting principles for observational studies and TRIPOD, TRIPOD+AI, and PROBAST principles relevant to prediction-model reporting and risk-of-bias assessment [20,21,22,23,24]. The study used a complete-case analytic dataset derived from 723 primary TKA records; 23 records were excluded before analysis because key radiographic alignment data and/or 12-month PROMs were incomplete. The participant flow is summarized in Supplementary Figure S1.

### 2.2. Participants and Eligibility Criteria

Eligible patients were consecutive adults who underwent primary TKA at the study center and had sufficient data to evaluate 12-month recovery. Inclusion criteria were (1) primary TKA for knee osteoarthritis; (2) availability of baseline standing radiographic alignment measurements; (3) availability of 10–14-month postoperative standing radiographic alignment measurements; and (4) availability of baseline and 12-month OKS and WOMAC PROMs.

Exclusion criteria were defined before analysis and were limited to data completeness and eligibility for the planned complete-case analysis: (1) incomplete key radiographic alignment variables; (2) unavailable baseline or 12-month PROMs; and (3) records not meeting the primary TKA source-frame requirement. Of 723 primary TKA records assessed for eligibility, 23 were excluded because of incomplete key radiographic alignment data and/or unavailable 12-month PROMs, leaving 700 complete cases for analysis (Supplementary Figure S1).

No additional post hoc exclusions were applied after the complete-case–cohort was defined. The exclusion count in the STROBE flow diagram therefore refers to records removed before final analysis, not to exclusions introduced during model fitting.

### 2.3. Outcome Definition

The primary endpoint was a strict composite MCID-based poor-recovery endpoint at 12 months. This endpoint was defined as failure to achieve the prespecified MCID for either OKS or WOMAC. This definition was intentionally conservative because persistent clinically important limitation in either an OKS-based knee-specific domain or a WOMAC-based pain/function domain may be relevant after TKA; however, it also increases the event rate and should not be interpreted as failure on both instruments. OKS improvement was calculated as 12-month OKS minus baseline OKS. WOMAC improvement was calculated as 12-month WOMAC minus baseline WOMAC; because lower WOMAC scores indicate fewer symptoms, more negative values indicate improvement. Component outcome definitions were OKS MCID failure (ΔOKS < 5) and WOMAC MCID failure (ΔWOMAC > −4), based on published MCID estimates after TKA [25,26,27,28]. These component endpoints and absolute 12-month thresholds were retained as sensitivity analyses to show how the strict composite definition influenced event frequencies.

As an additional outcome-definition sensitivity check, we calculated a more restrictive “AND” endpoint requiring failure to achieve both the OKS and WOMAC MCID thresholds. This endpoint was present in 209/700 patients (29.9%), confirming that the primary “either-measure” composite endpoint is conservative and yields a substantially higher event rate than either component alone or the dual-failure definition.

### 2.4. Predictor Timing and Leakage Control

Predictors were separated by measurement timing before analysis. The baseline-only model used age, sex, body mass index (BMI), preoperative pelvic tilt (PT), sacral slope (SS), lumbar lordosis (LL), hip–knee–ankle angle (HKA), spinopelvic index (SPI), baseline OKS, and baseline WOMAC. The contemporaneous model used the same baseline variables plus ΔPT, ΔSS, ΔLL, and ΔHKA. These change variables were measured at the 12-month follow-up window and therefore were not treated as prospective predictors of future outcomes. Knee flexion angle, psychosocial variables, spinal comorbidity, imaging modality, and surgical technical variables were not available in the analytic dataset and were therefore not imputed.

### 2.5. Phenotype Derivation

Spinopelvic compensation patterns were derived using k-means clustering with k = 3 on standardized PT, SS, LL, HKA, SPI, ΔPT, ΔSS, ΔLL, and ΔHKA. The three-cluster solution was retained as a clinically motivated exploratory structure rather than as proof of a naturally stable taxonomy. Because ΔPT, ΔSS, ΔLL, and ΔHKA were included, the resulting groups are not preoperative phenotypes. For descriptive analyses, clustering was performed in the complete analytic dataset, and groups were ordered by increasing strict composite endpoint prevalence. For cross-validated modeling, clustering was learned within training folds and then applied to held-out folds to reduce information leakage. Analyses were implemented in Python 3.10.12. using scikit-learn [29].

No claim is made that k = 3 represents the statistically optimal or externally stable number of clusters. The three-cluster solution is used as an interpretable exploratory structure; formal silhouette, elbow, bootstrap-stability, and external-validation analyses are identified as necessary next steps before these groups can be considered reproducible clinical phenotypes.

### 2.6. Model Development and Internal Validation

Ridge-penalized logistic regression was selected as the primary transparent model. Gradient boosting was evaluated as a non-linear comparator. Five-fold stratified cross-validation was used to generate out-of-fold predicted probabilities. Apparent performance was reported for context, but out-of-fold estimates were treated as the primary internal estimates. No external validation or independent cluster-replication cohort was available.

### 2.7. Performance, Calibration, and Clinical Utility

Discrimination was assessed with the area under the receiver operating characteristic curve (AUC). The Brier score was used to assess overall accuracy. Calibration was assessed by fitting a logistic calibration model in which the observed outcome was regressed on the logit of the predicted probability; the resulting intercept and slope are reported [30,31,32]. Logistic recalibration was used for exploratory decision curve analysis. Because the higher-performing model included 12-month alignment-change variables, decision curve results were interpreted only as hypothesis-generating evidence for contemporaneous postoperative stratification, not as evidence for preoperative decision-making [33].

### 2.8. Statistical Analysis

Continuous variables are summarized as mean ± standard deviation, and categorical variables as numbers and percentages. Pearson correlations were used to describe associations between alignment changes and PROM changes. The analysis is exploratory and internally validated only; no external validation cohort was available.

## 3. Results

### 3.1. Cohort and Outcome Frequency

The participant flow is shown in Supplementary Figure S1. Among 723 primary TKA records assessed for eligibility, 23 were excluded because of incomplete key radiographic alignment data and/or unavailable 12-month PROMs. The final complete-case analytic cohort included 700 patients. Mean age was 66.1 ± 8.1 years, 481/700 patients (68.7%) were female, and mean BMI was 28.3 ± 3.9 kg/m^2^. The strict composite MCID endpoint occurred in 537/700 patients (76.7%). Baseline demographics and PROMs by exploratory compensation pattern are shown in Table 1.

### 3.2. Exploratory Compensation Pattern Structure

**Figure 1:**
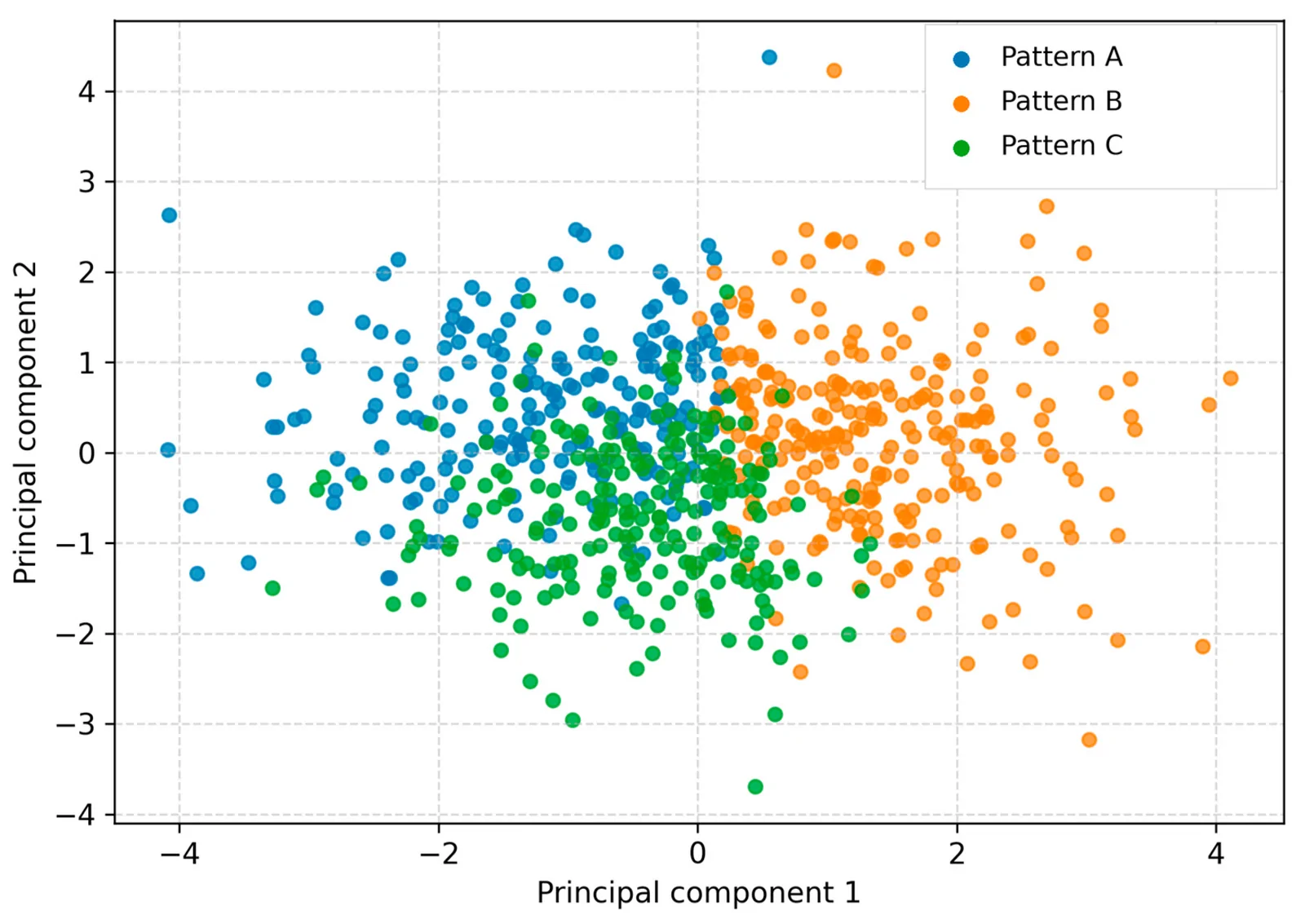
PCA visualization of exploratory spinopelvic compensation patterns derived by k-means clustering. Each point represents one patient projected onto the first two principal components computed from standardized alignment variables. PCA was used for visualization only. Pattern labels were ordered by increasing strict composite MCID endpoint prevalence.

**Figure 2:**
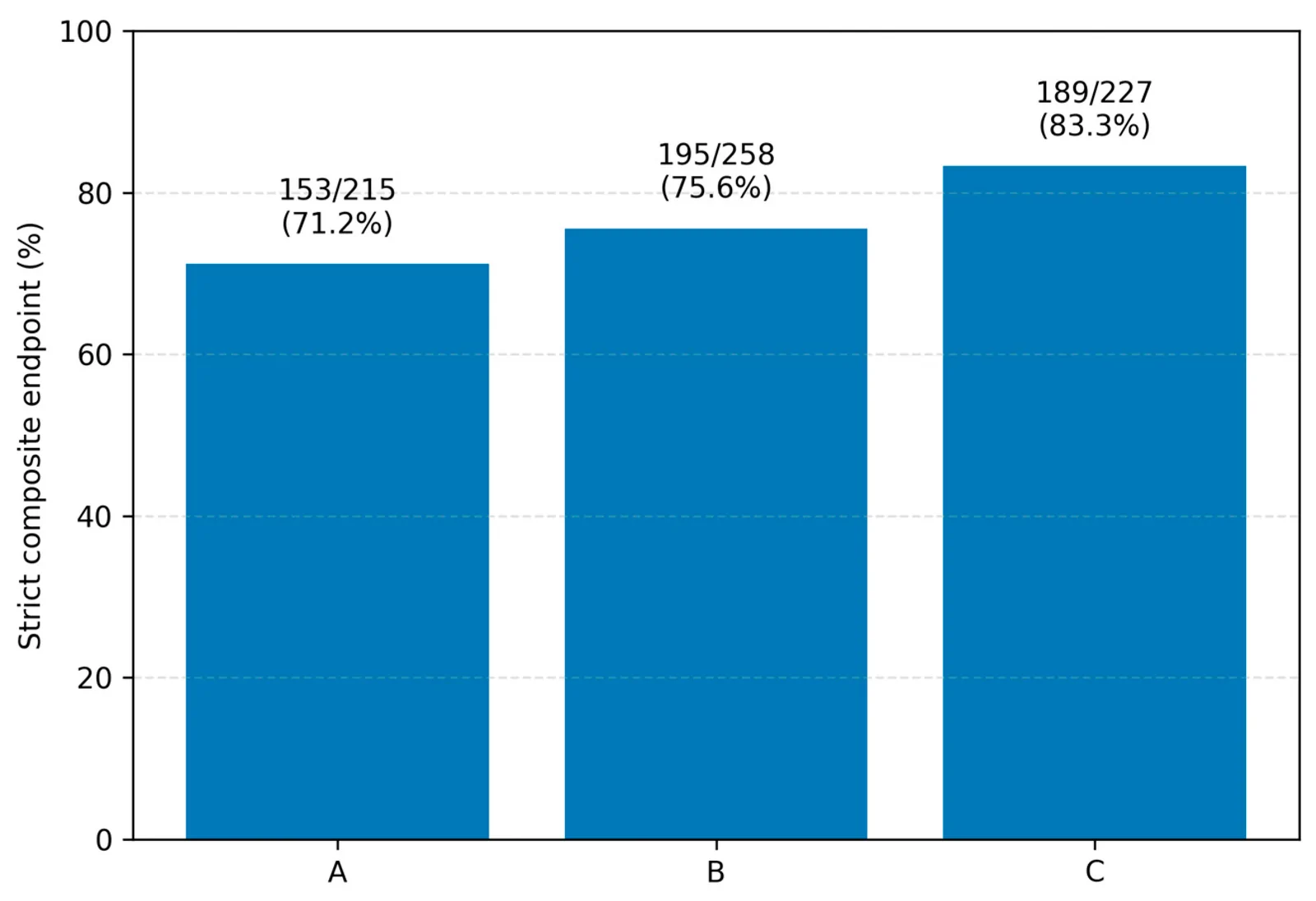
Strict composite MCID endpoint prevalence by exploratory compensation pattern. Bars show the percentage of patients who failed to achieve the prespecified MCID for either OKS or WOMAC at 12 months. Event counts and denominators are shown above each bar.

Three exploratory compensation patterns were identified. PCA visualization showed partial separation of the pattern structures in low-dimensional space (Figure 1). Strict composite endpoint prevalence increased from Pattern A to Pattern C: 153/215 (71.2%) in Pattern A, 195/258 (75.6%) in Pattern B, and 189/227 (83.3%) in Pattern C (Figure 2). Spinopelvic alignment characteristics by pattern are shown in Table 2, and lower-limb alignment and PROM changes are shown in Table 3.

For clinical readability, the patterns may be described as follows: Pattern A showed a higher pelvic tilt/lower lordosis profile with the largest PT reduction; Pattern B showed a higher sacral slope and higher lordosis profile with greater lumbar–sacral change; and Pattern C showed a lower sacral slope profile with more limited pelvic change and the highest strict composite endpoint prevalence. These labels are descriptive only and are not intended to define diagnostic categories.

### 3.3. Pattern C Interpretation

Pattern C had the highest strict composite endpoint prevalence and the least favorable mean WOMAC improvement (ΔWOMAC −2.73 ± 3.36), despite broadly similar baseline demographics and PROMs (Table 1 and Table 3). This finding should be interpreted cautiously because the patterns were internally derived in a single-center complete-case dataset and may reflect a combination of limited alignment adaptation, baseline-score effects, and unmeasured clinical heterogeneity rather than an externally transportable risk category.

### 3.4. Alignment Change and PROM Associations

**Figure 3:**
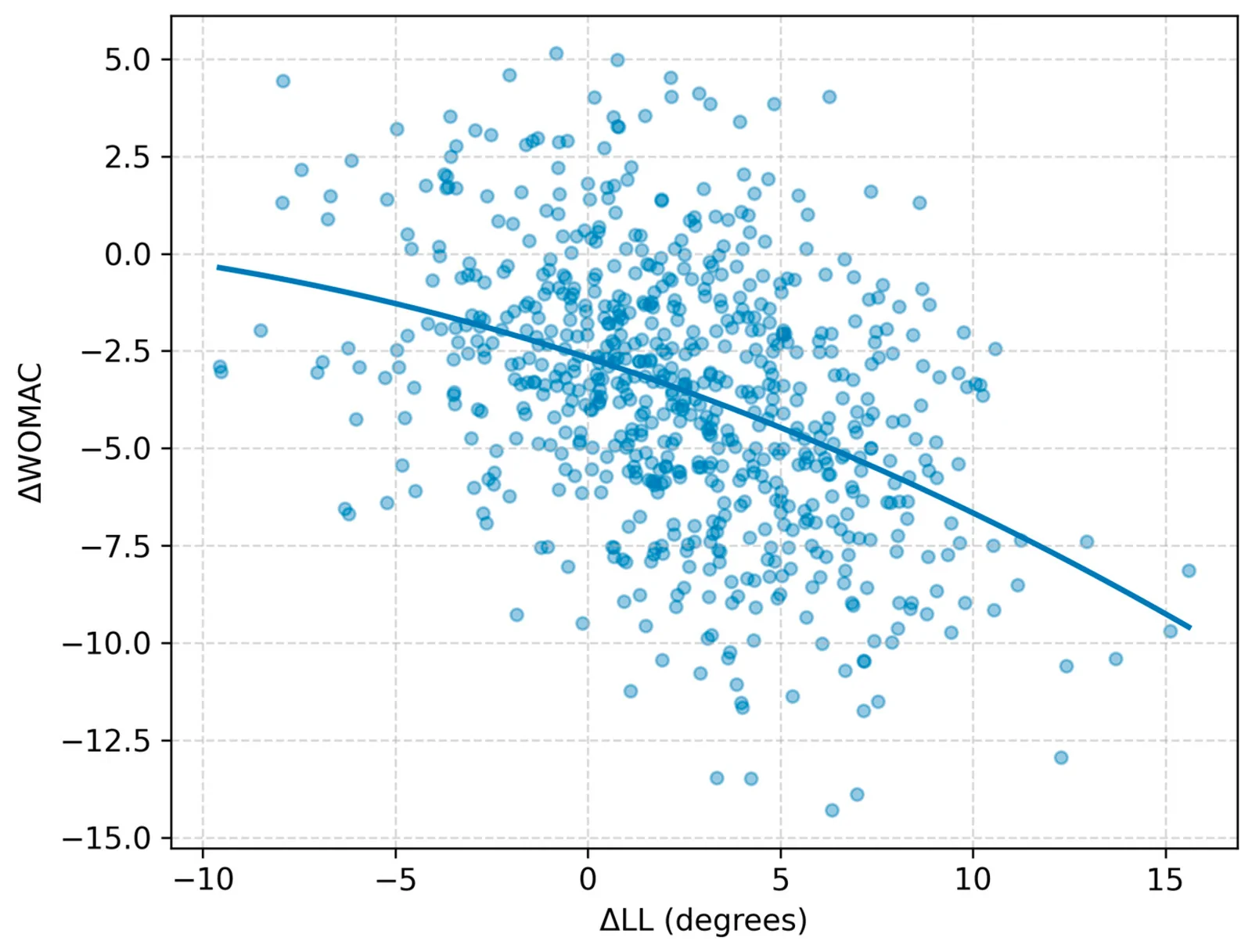
Exploratory association between change in lumbar lordosis (ΔLL) and WOMAC change. Each point represents one patient; the solid line summarizes the smoothed mean trend. Negative ΔWOMAC indicates symptom improvement because lower WOMAC scores reflect fewer symptoms. The relationship is descriptive and does not establish a causal effect of lumbar adaptation.

Alignment-change correlations with PROM improvements were modest to moderate (Table 4). ΔPT correlated with ΔOKS (r = −0.162, *p* < 0.001) and ΔWOMAC (r = 0.461, *p* < 0.001). ΔLL correlated weakly with ΔOKS (r = 0.082, *p* = 0.029) and moderately with ΔWOMAC (r = −0.403, *p* < 0.001). The exploratory ΔLL–ΔWOMAC association is shown in Figure 3. These findings support association-based interpretation but do not establish a causal biomechanical mechanism.

Although several correlations reached statistical significance, their clinical interpretation should be made with caution. The OKS correlations were weak and explained only a small proportion of outcome variance, while the WOMAC correlations were moderate but still insufficient to support a deterministic biomechanical explanation.

### 3.5. Model Performance, Calibration, and Clinical Interpretation

**Figure 4:**
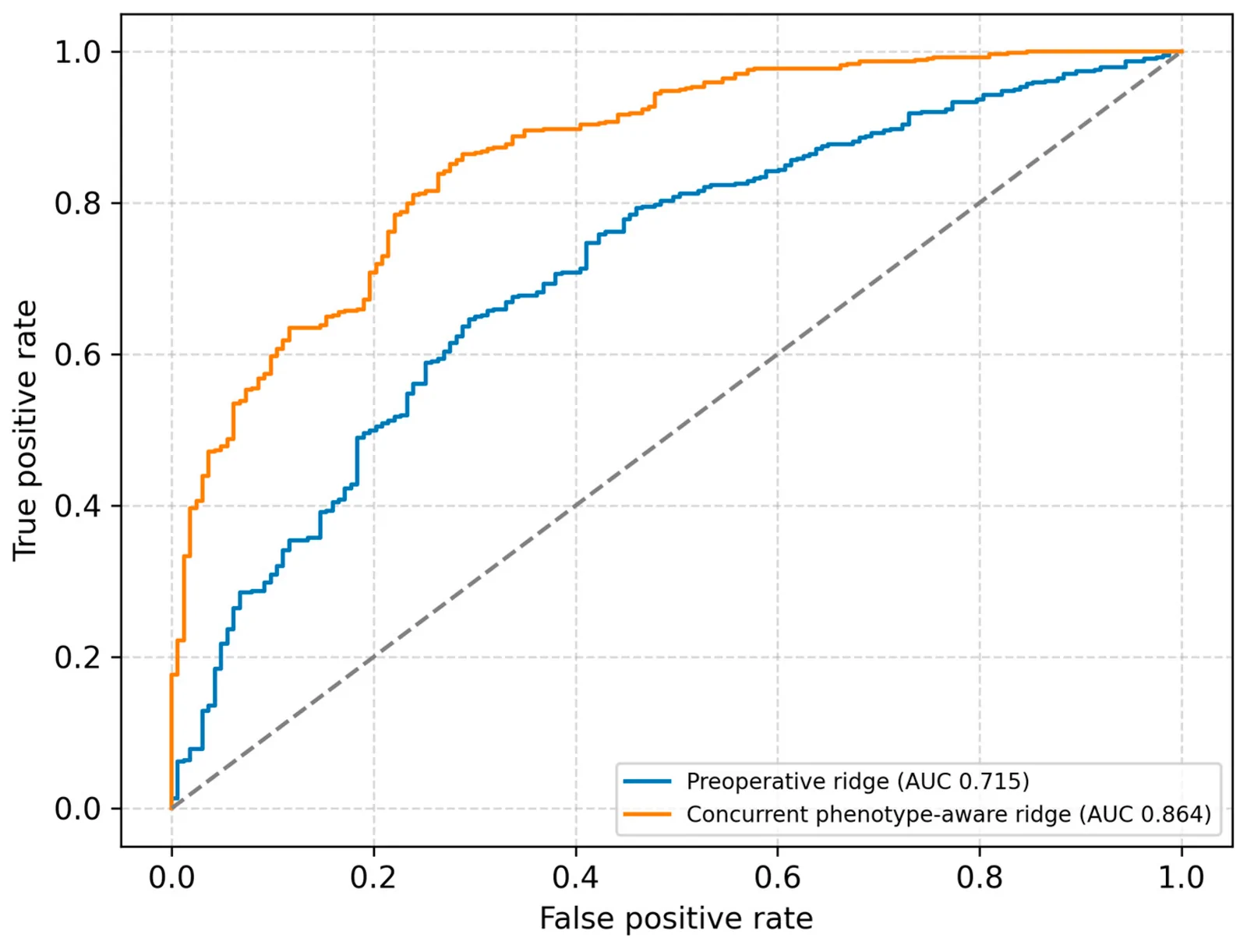
Out-of-fold receiver operating characteristic curves for the baseline-only ridge logistic model and the contemporaneous pattern-aware ridge logistic model. The baseline-only model used preoperative variables only. The contemporaneous model additionally included 12-month alignment-change variables. The dashed diagonal line represents the line of no discrimination (chance performance, AUC = 0.50).

**Figure 5:**
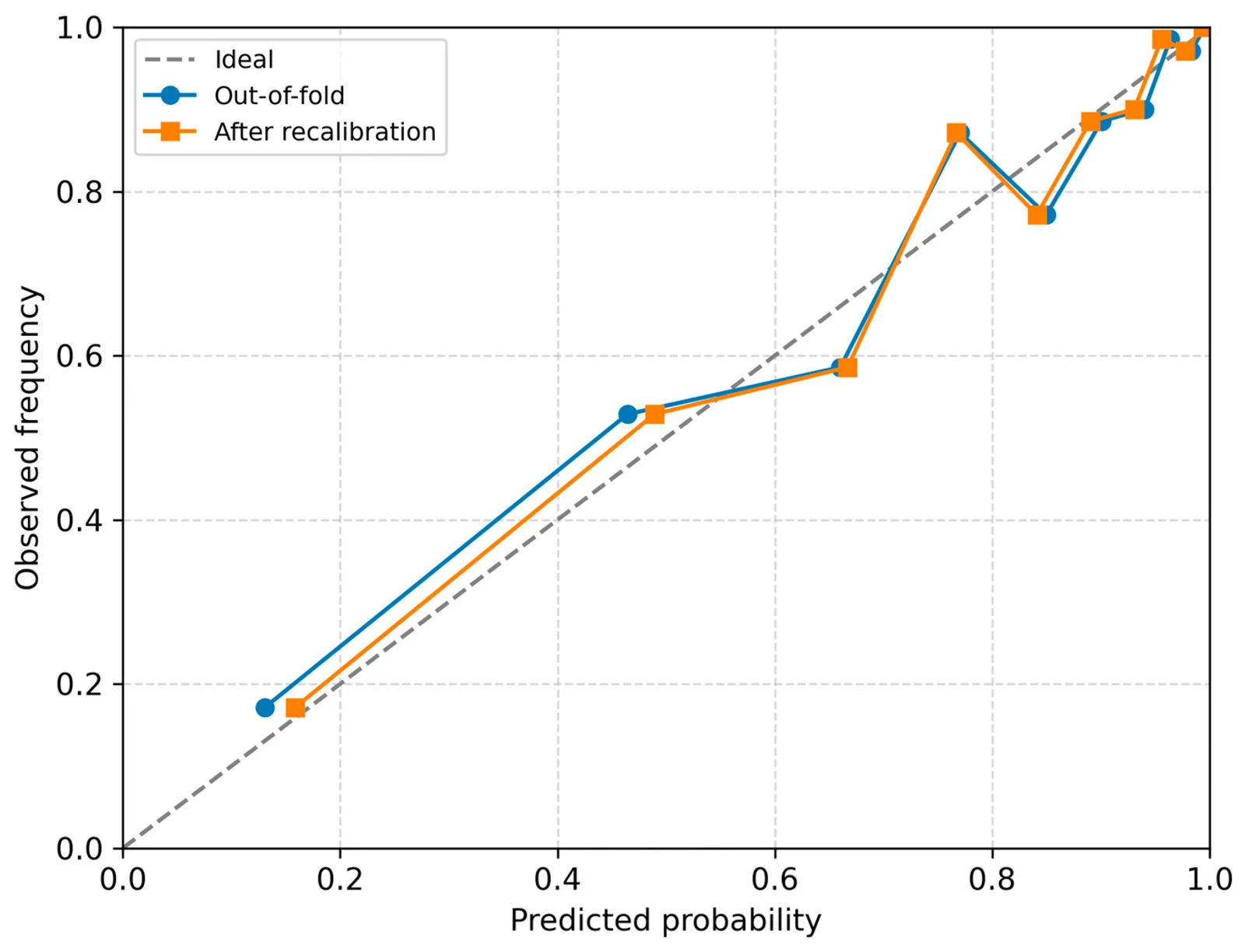
Calibration plot for the contemporaneous pattern-aware ridge logistic model using out-of-fold probabilities before and after logistic recalibration. Predicted probabilities are plotted against observed event frequencies; the diagonal line indicates perfect calibration. Recalibration was performed using the out-of-fold predicted probabilities to avoid an apparent-only calibration assessment.

**Figure 6:**
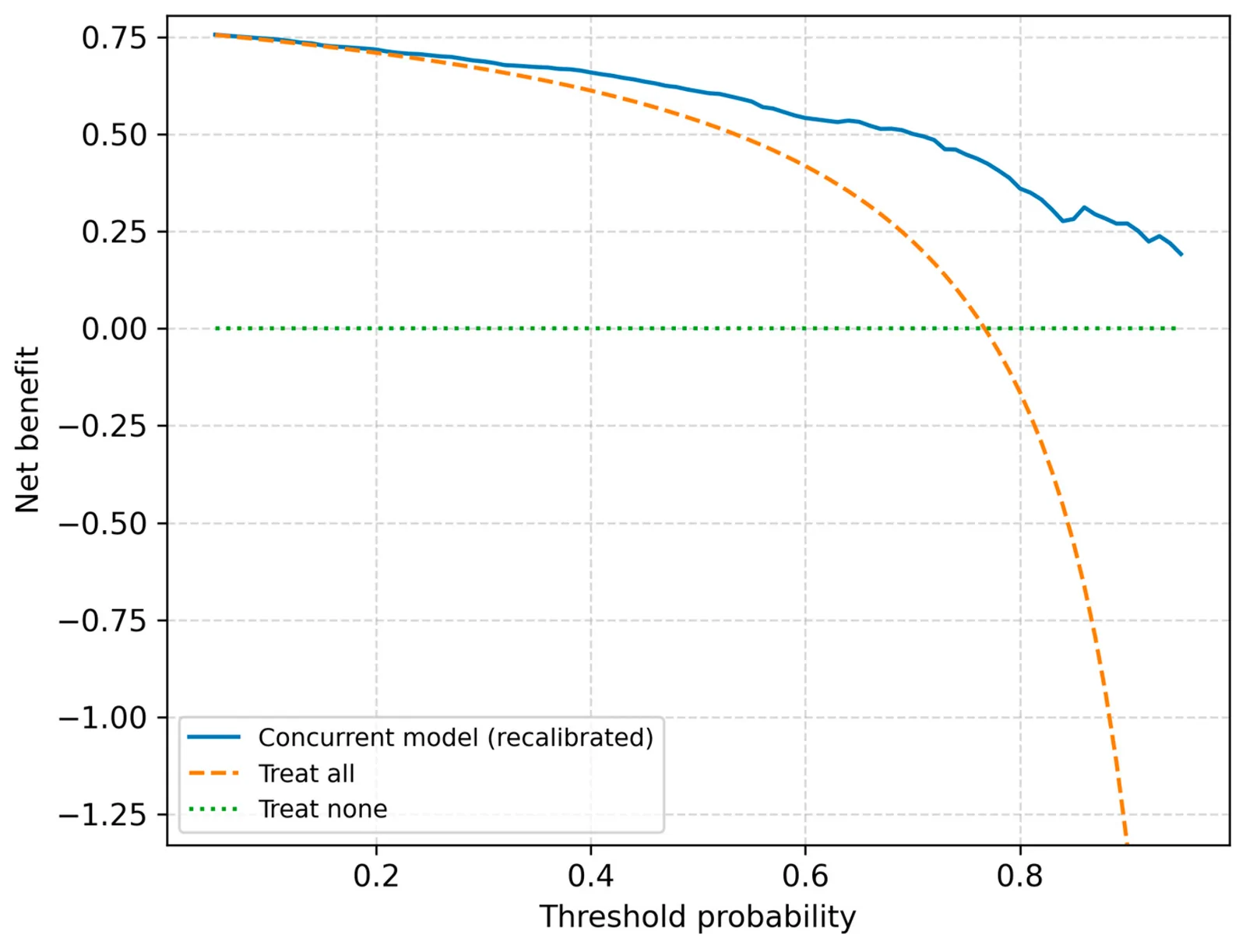
Decision curve analysis using recalibrated out-of-fold probabilities from the contemporaneous pattern-aware ridge logistic model. Net benefit is shown across threshold probabilities and is compared with treat-all and treat-none strategies. The curve should be interpreted as exploratory evidence for contemporaneous postoperative stratification only.

Internal model performance is summarized in Table 5. The baseline-only ridge logistic model achieved an out-of-fold AUC of 0.715 (95% CI 0.670–0.758), with a Brier score of 0.161 and a calibration slope of 0.884. The contemporaneous pattern-aware ridge model achieved higher internal discrimination, with out-of-fold AUC 0.864 (95% CI 0.834–0.894), Brier score 0.114, and calibration slope 0.909. ROC curves for the baseline-only and contemporaneous models are shown in Figure 4. Calibration before and after logistic recalibration is shown in Figure 5. Decision curve analysis using recalibrated out-of-fold probabilities is shown in Figure 6.

### 3.6. Explainability and Sensitivity Analyses

**Figure 7:**
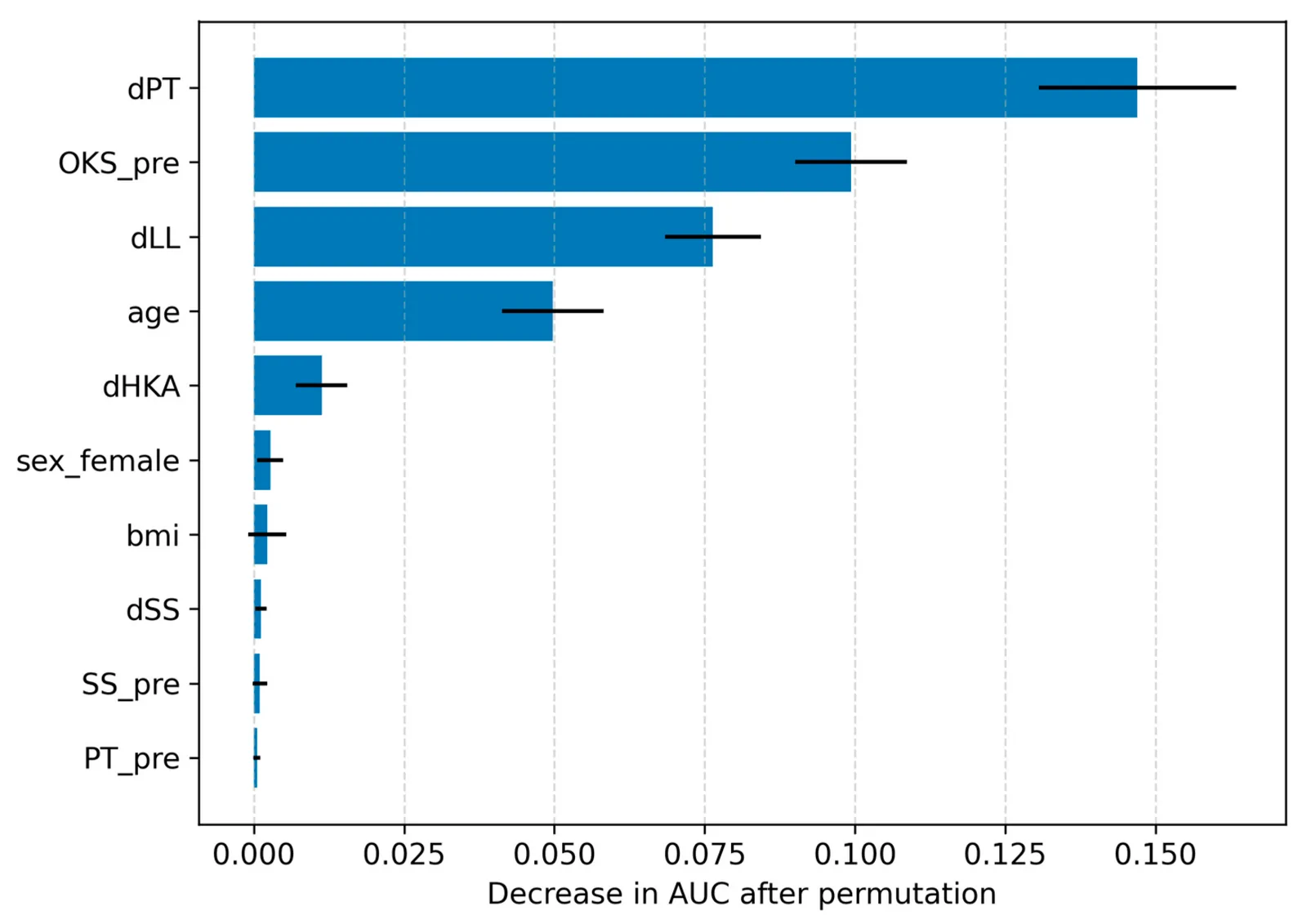
Global permutation importance for the contemporaneous ridge logistic model. Bars show the decrease in AUC after feature permutation, averaged across held-out folds. Alignment-change variables were measured during the 10–14-month postoperative window.

Permutation importance for the contemporaneous ridge model is shown in Figure 7. Baseline OKS was the dominant contributor, while alignment-change variables such as ΔLL and ΔPT contributed less strongly. Sensitivity analyses showed that component MCID outcomes had lower event rates than the strict composite endpoint: OKS MCID failure occurred in 350 patients (50.0%), WOMAC MCID failure in 396 patients (56.6%), and the strict composite endpoint in 537 patients (76.7%) (Table 6). This difference confirms that the composite definition is a conservative endpoint and should be interpreted accordingly.

Clinically, the feature ranking suggests that preoperative symptom burden/functional reserve, represented by baseline OKS, dominates the model, whereas ΔPT and ΔLL may reflect postoperative sagittal-chain adaptation. These importance values should be interpreted as model-dependent associations rather than causal effects or independent clinical treatment targets.

## 4. Discussion

This study supports a cautious interpretation of spinopelvic compensation patterns after TKA. The main finding is that baseline-only prediction of the strict composite 12-month MCID endpoint was modest, whereas stronger performance was observed only when the model incorporated alignment changes measured during the same follow-up window as the outcome. Accordingly, the higher-performing model should be interpreted as a contemporaneous association model rather than as a prospective clinical prediction tool.

Accordingly, the revised interpretation emphasizes three linked messages: the strict composite endpoint is conservative; the compensation patterns are internally derived exploratory constructs; and the higher-performing model is contemporaneous rather than a tool for preoperative decision-making.

The separation of predictor timing is central to interpretation. The baseline-only model used variables available before the 12-month follow-up window and achieved moderate internal discrimination. In contrast, the model that included ΔPT, ΔSS, ΔLL, and ΔHKA achieved higher discrimination but cannot support preoperative individual-level decisions because those variables are measured at postoperative follow-up.

Calibration was evaluated as a core performance domain. The primary estimates used out-of-fold predictions rather than apparent probabilities alone. Calibration slope was calculated using the standard logistic calibration approach with the logit of predicted risk as the independent variable. Although recalibration improved graphical agreement, external validation and model updating remain necessary before clinical implementation.

The exploratory pattern findings are clinically suggestive but not definitive. The increasingly strict composite endpoint gradient across Patterns A to C suggests that spinopelvic compensation patterns may capture recovery heterogeneity. However, these internally derived groups should not be treated as validated phenotypes until replicated with formal cluster-stability testing and independent external cohorts.

The endpoint definition also requires cautious interpretation. Because failure to reach the MCID on either OKS or WOMAC classified a patient as meeting the strict composite endpoint, the event rate was higher than that for either component outcome alone. The component-outcome sensitivity analyses were therefore included to make this consequence transparent and to avoid overstating the proportion of patients with globally poor recovery.

Clinically, the results are best viewed as a basis for future research on spine-aware arthroplasty pathways. A validated baseline-only model could eventually support preoperative counseling and follow-up planning. By contrast, contemporaneous stratification may help characterize patients with limited 12-month recovery and guide hypotheses for postoperative rehabilitation or further musculoskeletal evaluation. External validation, model updating, and transportability assessment are required before implementation [34,35,36].

## 5. Limitations

This study has several limitations. It was retrospective and single-center in design, and external validation was not available. Although the analytic flow is explicit (723 records assessed, 23 excluded, and 700 included), detailed subcategories for the 23 exclusions were not available; therefore, the exclusion box is reported conservatively as having incomplete key radiographic alignment data and/or unavailable 12-month PROMs.

Selection bias remains possible because the analysis used complete cases only. Although only 23 of 723 records were excluded, detailed subcategories and complete baseline comparisons for excluded patients were not available, so systematic differences between included and excluded patients cannot be ruled out. These factors may limit generalizability to other centers, imaging protocols, surgical workflows, rehabilitation pathways, and healthcare systems.

The strict composite MCID endpoint may be influenced by baseline PROM status and may produce a higher event rate than either OKS or WOMAC MCID failure alone. Component outcomes and absolute 12-month status thresholds were therefore reported as sensitivity analyses, but these analyses do not remove all baseline-score dependence. The dataset also lacked knee flexion angle, psychosocial measures, spinal comorbidity, imaging modality, and detailed surgical variables, all of which may influence TKA recovery.

The contemporaneous model includes ΔLL, ΔPT, ΔSS, and ΔHKA measured during the same follow-up window as PROM outcomes; therefore, it supports association-based stratification but not prospective prediction. Finally, the exploratory compensation patterns were internally derived, included postoperative change variables, and required formal stability assessment and independent external validation before they can be considered clinically stable phenotypes.

## 6. Conclusions

Baseline-only prediction of the strict composite 12-month MCID endpoint after TKA was modest. Stronger model performance was observed only in a contemporaneous association model that incorporated 12-month alignment-change variables. Exploratory spinopelvic compensation patterns were associated with endpoint prevalence, but they should not be considered validated clinical phenotypes or used for individual-level decisions without external validation.

## Figures and Tables

**Table 1:** Baseline demographics, preoperative PROMs, and strict composite MCID endpoint frequency by exploratory compensation pattern.

Pattern	*n*	Female, *n* (%)	Age, Years	BMI, kg/m^2^	OKS_pre	WOMAC_pre	Strict Composite MCID Endpoint, *n* (%)
A	215	149 (69.3%)	66.36 ± 7.63	28.23 ± 4.09	21.48 ± 6.37	59.38 ± 12.39	153 (71.2%)
B	258	177 (68.6%)	66.52 ± 8.51	28.09 ± 3.63	22.24 ± 6.46	61.10 ± 12.40	195 (75.6%)
C	227	155 (68.3%)	65.33 ± 7.90	28.48 ± 4.08	21.26 ± 6.18	61.49 ± 11.68	189 (83.3%)

Note. Values are presented as mean ± SD unless otherwise stated. The endpoint was defined as failure to achieve the prespecified MCID for either OKS or WOMAC at 12 months. Abbreviations: BMI, body mass index; OKS, Oxford Knee Score; WOMAC, Western Ontario and McMaster Universities Osteoarthritis Index; MCID, minimal clinically important difference.

**Table 2:** Preoperative, 12-month postoperative, and change values for spinopelvic alignment by exploratory compensation patterns.

Pattern	PT Pre/Post/Δ	SS Pre/Post/Δ	LL Pre/Post/Δ
A	27.13 ± 5.82/21.79 ± 7.29/−5.34 ± 3.16	36.82 ± 6.53/38.86 ± 7.20/2.04 ± 3.01	42.65 ± 7.81/44.54 ± 9.31/1.89 ± 3.96
B	18.67 ± 6.54/14.74 ± 7.07/−3.96 ± 3.26	41.75 ± 6.33/45.02 ± 6.87/3.27 ± 2.85	55.78 ± 9.02/58.88 ± 10.33/3.10 ± 3.92
C	19.71 ± 5.83/17.68 ± 7.09/−2.05 ± 3.01	29.67 ± 5.50/32.44 ± 6.23/2.76 ± 2.94	49.52 ± 8.09/51.65 ± 8.29/2.13 ± 3.55

Note. Values are presented as mean ± SD. Each cell shows preoperative/postoperative/change values. Δ denotes 12-month postoperative minus preoperative measurement. Abbreviations: PT, pelvic tilt; SS, sacral slope; LL, lumbar lordosis.

**Table 3:** Lower-limb alignment and patient-reported outcome changes by exploratory compensation pattern.

Pattern	HKA Pre/Post/Δ	ΔOKS	ΔWOMAC
A	5.61 ± 2.39/2.33 ± 2.45/−3.81 ± 1.87	6.21 ± 4.06	−4.11 ± 3.38
B	6.00 ± 2.63/2.81 ± 2.68/−3.65 ± 1.86	5.69 ± 4.16	−3.96 ± 3.31
C	5.56 ± 2.41/2.50 ± 2.52/−3.47 ± 1.94	5.74 ± 4.10	−2.73 ± 3.36

Note. Values are mean ± SD. HKA cells show preoperative/postoperative/change values; PROM columns show 12-month postoperative minus baseline change. Negative ΔWOMAC indicates symptom improvement because lower WOMAC scores reflect fewer symptoms. Abbreviations: HKA, hip–knee–ankle angle; OKS, Oxford Knee Score; WOMAC, Western Ontario and McMaster Universities Osteoarthritis Index.

**Table 4:** Correlations between changes in alignment and changes in patient-reported outcomes.

Pair	Pearson r	*p* Value
ΔPT vs. ΔOKS	−0.162	<0.001
ΔPT vs. ΔWOMAC	0.461	<0.001
ΔSS vs. ΔOKS	0.022	0.562
ΔSS vs. ΔWOMAC	−0.02	0.598
ΔLL vs. ΔOKS	0.082	0.029
ΔLL vs. ΔWOMAC	−0.403	<0.001
ΔHKA vs. ΔOKS	−0.02	0.590
ΔHKA vs. ΔWOMAC	0.213	<0.001

Note. Correlations are Pearson r. Δ denotes 12-month postoperative minus preoperative measurement for alignment variables and 12-month minus baseline for PROMs. Negative ΔWOMAC indicates symptom improvement. Correlations are descriptive and do not imply a causal mechanism.

**Table 5:** Internal model performance and calibration metrics.

Model	Predictor Timing	Apparent AUC	Out-of-Fold AUC (95% CI)	Brier	Calibration Slope
Preoperative ridge logistic	Preoperative only	0.735	0.715 (0.670–0.758)	0.161	0.884
Preoperative gradient boosting	Preoperative only	0.874	0.716 (0.668–0.758)	0.161	0.813
Contemporaneous pattern-aware ridge logistic	Preoperative + 12-month alignment change + fold-derived pattern	0.882	0.864 (0.834–0.894)	0.114	0.909
Contemporaneous gradient boosting	Preoperative + 12-month alignment change	0.954	0.871 (0.840–0.899)	0.114	1.264

Note. Out-of-fold estimates are the primary internal performance estimates. Apparent AUC is shown only for context. The baseline-only model used only preoperative variables; the contemporaneous model additionally used 12-month alignment-change variables and a fold-derived exploratory compensation pattern. Calibration slope was calculated by regressing the observed outcome on the logit of out-of-fold predicted probability.

**Table 6:** Outcome definition sensitivity analyses for the strict composite MCID endpoint.

Outcome Definition	Events, *n* (%)
OKS MCID failure only (ΔOKS < 5)	350 (50.0%)
WOMAC MCID failure only (ΔWOMAC > −4)	396 (56.6%)
Strict composite MCID endpoint	537 (76.7%)
Absolute 12-month poor OKS (OKS ≤ 25)	364 (52.0%)
Absolute 12-month poor WOMAC (WOMAC ≥ 50)	507 (72.4%)
Dual OKS and WOMAC MCID failure (AND endpoint)	209 (29.9%)

Note. OKS MCID failure and WOMAC MCID failure are component outcomes of the strict composite endpoint. Absolute 12-month thresholds are descriptive sensitivity analyses only and were used to evaluate whether the composite MCID endpoint was sensitive to baseline PROM status.

## Data Availability

The data sets generated and/or analyzed during the current study are not publicly available because of institutional data-protection policies but are available from the corresponding author upon reasonable request.

## Supplementary Materials

The following supporting information can be downloaded at: https://www.mdpi.com/article/10.3390/medicina62081481/s1, Figure S1: STROBE-style participant flow diagram. A total of 723 primary TKA records were assessed for eligibility. Twenty-three records were excluded before analysis because of incomplete key radiographic alignment data and/or unavailable 12-month PROMs. The final complete-case analytic cohort comprised 700 patients. STROBE-style participant flow diagram showing 723 records assessed for eligibility, 23 exclusions for incomplete key radiographic alignment data and/or unavailable 12-month PROMs, and 700 complete cases included in the final analysis.
