# Supplementary material for: Exploratory Spinopelvic Compensation Patterns Are Associated with a Strict Composite 12-Month MCID Endpoint After Total Knee Arthroplasty: A Retrospective Risk-Stratification Study
Source: Medicina (Kaunas). 2026 Aug 1;62(8):1481. doi: 10.3390/medicina62081481 (PMC13514588; doi:10.3390/medicina62081481)
Supplement: Supplementary file 1 [file medicina-62-01481-s001.zip › medicina-4388574-supplementary.pdf]

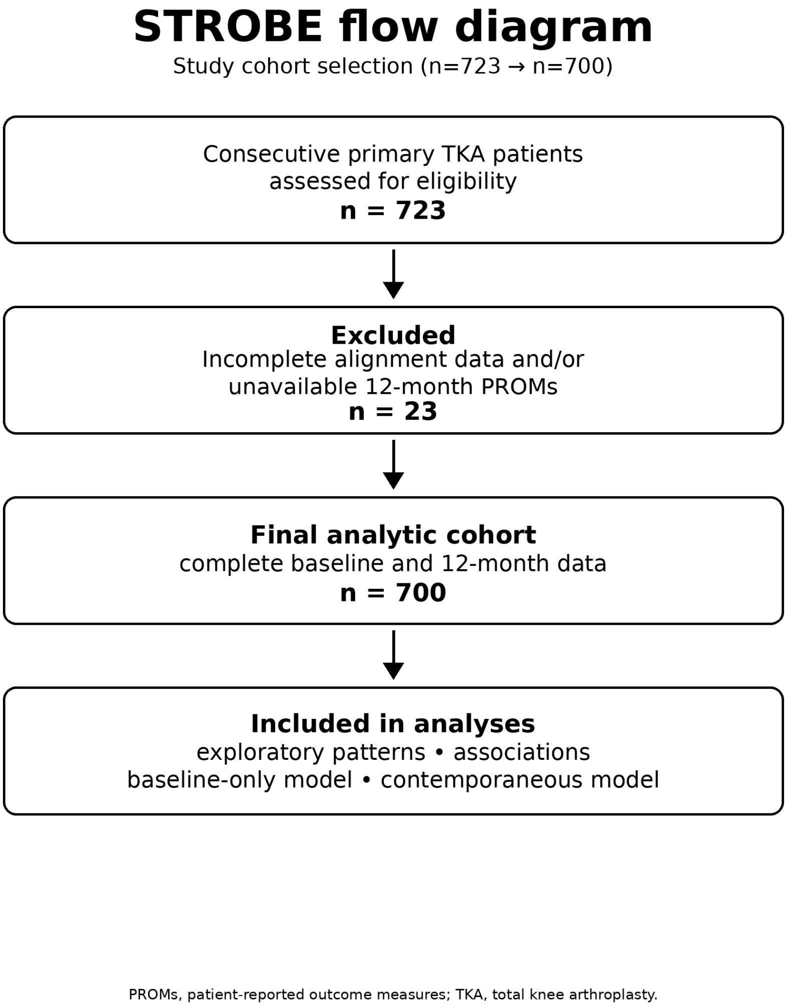

Supplementary Figure S1. STROBE-style participant flow diagram. A total of 723 primary TKA records were assessed for eligibility. Twenty-three records were excluded before analysis because of incomplete key radiographic alignment data **and/or** unavailable 12-month PROMs. The final complete-case analytic cohort comprised 700 patients. STROBE-style participant flow diagram showing 723 records assessed for eligibility, 23 exclusions for incomplete key radiographic alignment data and/or unavailable 12-month PROMs, and 700 complete cases included in the final analysis.
